# Supplementary figures and images for: Estimating the cumulative incidence of SARS-CoV-2 with imperfect serological tests: Exploiting cutoff-free approaches
Source: PLoS Comput Biol. 2021 Feb 26;17(2):e1008728. doi: 10.1371/journal.pcbi.1008728 (PMC7946301; doi:10.1371/journal.pcbi.1008728)

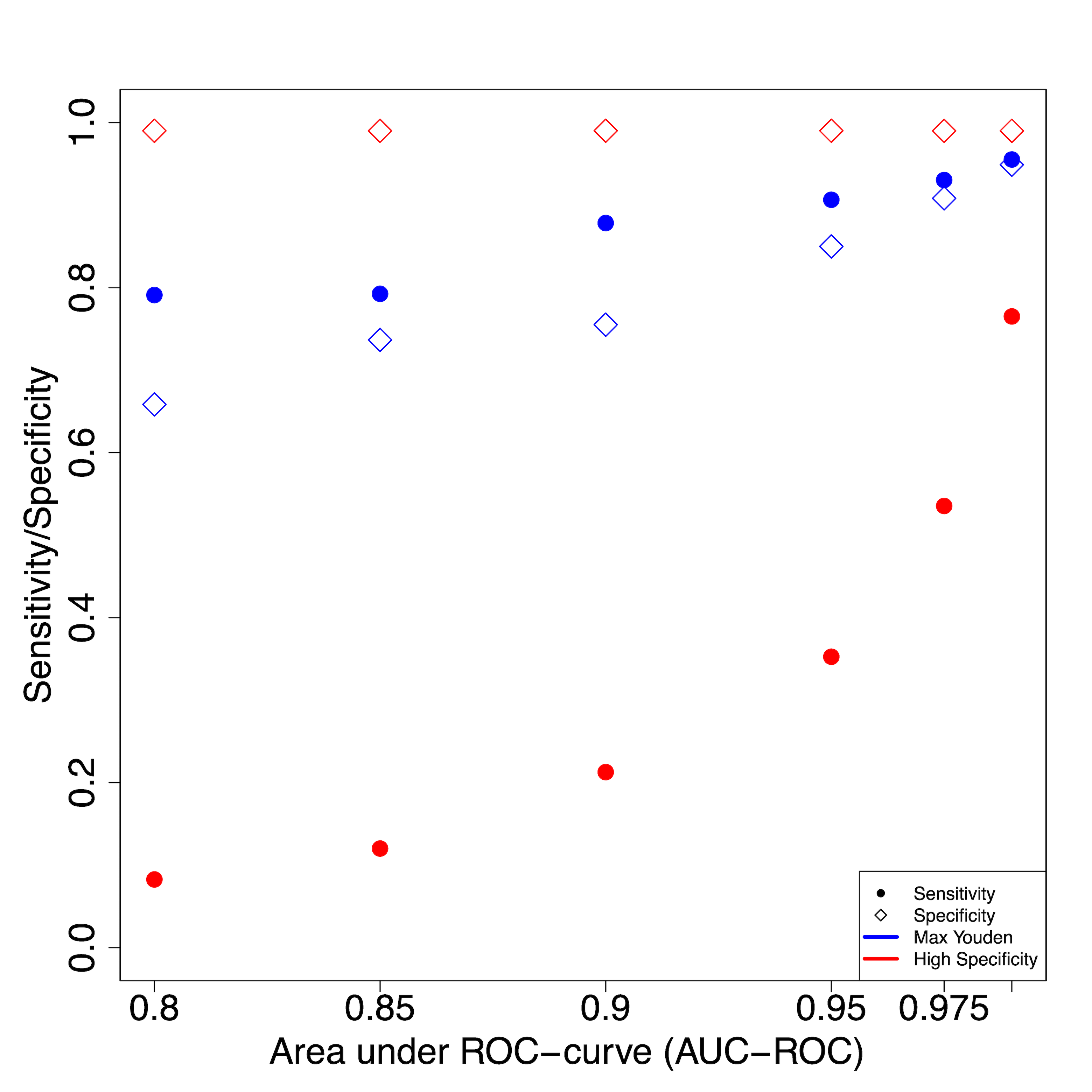

Supplement: S1 Fig — (TIF) [file pcbi.1008728.s001.tif]

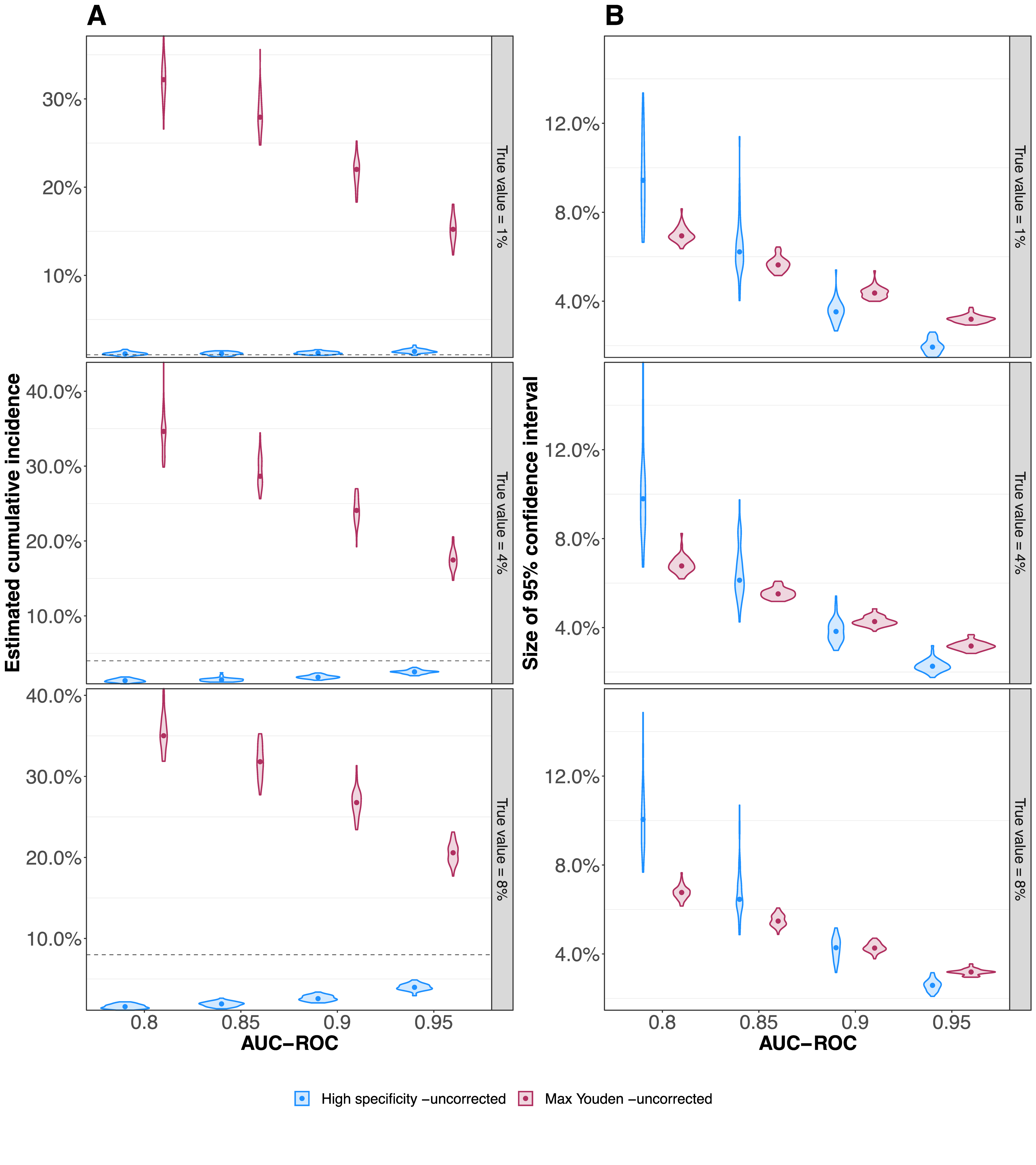

Supplement: S2 Fig — (A) Estimated cumulative incidence for three levels of true cumulative incidence. (B) Half of the 95% confidence intervals estimated based on the bootstrap method for both uncorrected cutoff-based methods. (TIF) [file pcbi.1008728.s002.tif]
