# Supplementary material for: Estimating the cumulative incidence of SARS-CoV-2 with imperfect serological tests: Exploiting cutoff-free approaches
Source: PLoS Comput Biol. 2021 Feb 26;17(2):e1008728. doi: 10.1371/journal.pcbi.1008728 (PMC7946301; doi:10.1371/journal.pcbi.1008728)
Supplement: Attachment — Submitted filename: Response_to_reviewers_JB_10_8-rrr.docx [file pcbi.1008728.s003.docx]

**Point-by-point replies to the Editor and the Reviewers**

We would like to thank the reviewers for the time and effort they have invested in reviewing our paper. We found their comments very insightful and believe that the revised manuscript represents a significant improvement over our original submission. Below, we provide point-by-point responses to the reviewers’ comments in blue. In red, we present relevant parts of the revised manuscript.

**Editors’ Comments to the Authors:**

We cannot make any decision about publication until we have seen the revised manuscript and your response to the reviewers' comments. In particular, Reviewer 2 raises a number of substantive methodological issues which will need to be addressed to their satisfaction. Reviewer 1 raises a question of novelty, but perhaps a full response to Reviewer 2's concerns would address this issue.

Author response: As you will see below, we addressed all methodological points carefully, and comprehensively. The point that was most difficult to address – because it required us to rethink the method and re-implement the mixture-model approach – was Reviewer #2’s concern that we have been using parametric gamma distributions to describe the distributions of quantitative test measures. In the revised paper, we now switched to using empirical distributions. While this forced us to completely revise our code, simulations and figures, it makes the method more flexible and widely applicable.

With respect to the novelty, we largely agree with Reviewer #1 that the mixture-model approach “is already well-established”. We were particularly happy to read though that we provide “an excellent reference on seroprevalence estimation methods”. Indeed, there is not a single paper that comprehensively describes the mixture-models and compares their performance and potential with cutoff-based approaches.

We respectfully disagree, however, in the judgment of Reviewer #1 that ”The addition of a third "unknown" distribution within the data is a nice addition with a clear application, even if it is not a ground-breaking development”. In our view, detecting distributions in serosurvey data that have not been included in the validation of serological tests, such as mild or asymptomatic infections, is of utmost importance in the current SARS-CoV-2 pandemic. While statistically feasible and easy to conduct, such analyses have been lacking in the context of SARS-CoV-2 serosurveys. As a result, serosurvey data are not analyzed to the full extent of their potential. The approach we propose to detect and estimate the prevalence of infection types that have been left out during validation has the potential to fill our knowledge gap about the incidence of mild and asymptomatic infections.

**Reviewers’ Comments to the Authors:**

**Reviewer #1:**

The manuscript by Bouman and colleagues, entitled "Estimating seroprevalence with imperfect serological tests: exploiting cutoff-free approaches", presents an investigation of the performance of methods for estimating seroprevalence when data/assays are imperfect.

The main focus of the study is on the comparison of methods based on binary data (being antibody positive or not) and those that use quantitative antibody level data. They use a range of relevant simulations to assess how well different methods work, and show that a likelihood-based mixture model performs particularly well, especially when test specificity and sensitivity are lower.

The study provides an excellent reference on seroprevalence estimation methods, investigates highly relevant aspects in a careful way, is well written and easy to follow. The authors also helpfully provided R functions that help implement the methods (although I wasn't able to try the functions because I couldn't access them in the online review system).

I don't have any major comments on how the study was conducted or presented.

Author response: We thank the reviewer for this summary. We are honored that our study is referred to as ´an excellent reference´. The R functions are available here: <https://gitlab.ethz.ch/jbouman/pist>.

The only reservation I might have is on the novelty aspect of the work. The main focus of the study is the use of quantitative vs binary antibody data for estimating seroprevalence, and the application of a likelihood-based mixture model approach.
This approach is already well-established as being particularly suitable in a context of imperfect data, and has been used frequently.
The authors clearly acknowledge this and cite relevant work.
The addition of a third "unknown" distribution within the data is a nice addition with a clear application, even if it is not a ground-breaking development.

So it seems that the real novelty, and purpose of this study, is in the formal comparison of the different methods, and in combining it with a power analysis and useful R functions.
When first reading the abstract and main text, I was expecting a fully new approach to this problem, and it took me a while before I realized this is not the purpose of the study. A suggestion to avoid this would be to rephrase it slightly in the abstract and introduction as a review of existing methods, and that the purpose of the study is rather to be a useful reference, as opposed to a new development in this field.

Author response: We agree with the reviewer that the mixture-model approach has been described previously, and we carefully revised our manuscript to avoid the impression that this method was developed by us. Throughout the revised manuscript we therefore refer to the method as the ‘mixture-model estimator/approach’ rather than the ‘likelihood-based method’ we use in the original manuscript. As ‘mixture model’ is also the term used for this method in previous literature, we believe this makes it more transparent that this method itself is not new.

We believe that our introduction, in particular paragraph 4 of the revised manuscript, describes clearly that the mixture-model approach has been used in previous work. In paragraph 5 and 6 of the revised manuscript, we make clear that the goal of this study is to compare this method to cutoff-based methods with the use of simulations and that we introduce an extended model that can detect a missing case distribution.

In contrast to the reviewer, we believe that this addition of a third ‘unknown’ distribution in the validation data is an important addition to the current literature. This method does not only prevent a significant bias in the overall cumulative incidence estimate, but also gives an estimate of what fraction of the infections is due to this additional distribution.

Minor comments:

- According to nomenclature guidelines coronavirus should not be capitalized.

Author response: We chosen to use SARS-CoV-2 consistently throughout the revised paper.

- The results in figure 1 are useful for comparison, but I wonder whether it would be useful to add more formal measures of the errors. Now I am for example left wondering whether or not the likelihood method outperforms the corrected Max Youden method.

Author response: We agree with this point. We have therefore added an extra panel to figure 1 that shows the 95% uncertainty intervals of all methods. This figure makes clear that the mixture model indeed outperforms the other methods in terms of uncertainty of the estimates.

- It might be helpful to provide more information in the figure legends so they can be understood more easily.

Author response: We have added more information to all figure legends.

- Axis tick label fonts are quite small (especially in fig 2), consider increasing.

Author response: We have increased label fonts to improve readability of the figures.

- I was a bit visually distracted by the grid lines in the figures, but that's probably a highly subjective preference.

Author response: Gridlines are removed from most figures.

- Throughout the text the number 10,000 was written with an inverted comma (10'000), is this a mistake or on purpose?

Author response: We have changed 10’000 into 10,000. (“10’000” is the Swiss spelling of ten thousand. We apologize that this Swissims slipped through.)

- I found a few typos but they are tricky to point out due to the lack of line numbers (suggestion: add line numbers whenever sending a document for review).

Author response: We apologize for not providing the line numbers. We carefully went through the manuscript to correct typos.

We thank the reviewer for pointing out these minor comments.

**Reviewer #2:**

Bouman et al have submitted a manuscript called "Estimating seroprevalence with imperfect serological tests: exploiting cutoff-free approaches" for consideration in PLOS Computational Biology. The manuscript is stimulating at present but is not currently publishable in PLOS Computational Biology, due to issues that are discussed below.

Overall, the argument of the manuscript is that the estimation of seroprevalence can be improved via the use of a cutoff-free / likelihood-based approach. The assumption is that the observed scalar data come from a mixture of two or more classes of signals (seropositive and seronegative, in the simplest case), and that each individual data point belongs to one of the classes. In other words, the authors use a one-dimension mixture model with K=2 (negative, positive) or K=3 (negative, mild positive, severe positive). In this context, to determine seroprevalence, one need only infer the mixture weight(s).

Author response: We thank the reviewer for this accurate summary of the core ideas of our study. At this place, we would also like to thank the reviewer for all the “broad comments”. We found them very insightful and sincerely believe they have improved our study significantly (irrespective of the outcome of our resubmission.

Broad Comments:

This manuscript's proposition rests on the establishment of two key claims:

1. The likelihood-based approach works.
2. The likelihood-based approach provides superior estimates.

The manuscript at present has not quite established those claims.

Author response: We appreciate the clear stating of the two main claims of our submitted manuscript. This made us realize that our writing did not convey the main claims that we wanted to make. Therefore, we rephrased the 5^th^ paragraph of the introduction and the discussion to clarify that the two main statements of our study are:

1. The likelihood-based (now ”mixture model”) approach works, i.e. the estimates are comparable to traditional estimates and result in smaller uncertainty intervals.
2. The likelihood-based method allows us to investigate the heterogeneity in disease outcomes that cannot be addressed by the cutoff-based methods.

1. That the likelihood-based approach works is not quite established in this paper due to the fact that it assumes (I think?) that continuous responses come from a known distribution. Here, for instance, it appears that OD values are gamma distributed in the simulations. What if gamma are used for inference, but the true values come from a different distribution? What if neither distribution is gamma? Is model selection to learn the class of data-generating distributions possible with sufficient data? What if, e.g., the true distribution of test measures for positive cases is a truncated normal distribution and a gamma distribution is selected for the model?

Author response: We thank the reviewer for pointing this out and we agree that this was indeed a weakness of the work in our original submission. In response, we have adapted our method to directly use the empirical distribution of the case and control sera of the validation data, instead of fitting a gamma distribution through the data. The details of this adaptation can be found in paragraph 3 of the section ‘Simulations’ of the methods.

In Figure 1 we compare our revised method (empirical) to the methods from the original manuscript where we assumed the true parameters of the case and control distribution to be known (gamma distribution with known mean and variance) and where we estimated the mean and variance from case and control data (fitted gamma distribution). We found that using the empirical distribution provides better estimates than inferring a gamma distribution (see Figure 1A and C). Therefore, we have decided to switch all of our study to empirical distributions.

Moreover, Figure 1B and D also show that indeed, the assumption of the gamma distribution leads to problems when the true distribution is a truncated normal. With empirical distributions the mixture-model method is robustly works for various true distributions.

Please note that due to this improvement of our method, we also had to change the way in which we apply the cutoff-based method to our simulated data. This is described in paragraph 3 of the section ‘Simulations’ of the methods. In summary, to keep the comparison of the methods fair, we used the same case and control sera of the validation data to estimate the sensitivity and specificity of the cutoff-based methods as we used to infer the empirical distributions for the mixture model.

Moreover, we have moved the paragraph that described the bootstrapping procedure of estimating the confidence interval from the results to the methods. It can now be found in the subsection ‘Confidence interval’ in the Methods section. The reason for this is that by changing the method, we now use the bootstrapped confidence interval for all the results we present.

Figure 1: Comparison of the revised method based on the empirical distribution of the control and case sera with the parametric approaches. The revised method is called ‘empirical’ and is shown in red, in green we show the estimates made while assuming the true mean and variance of the gamma distributions to be known and in blue we show estimates where a gamma distribution is fitted through the control and case sera. We show the estimated cumulative incidence of 50 simulations for each AUC-ROC value of the serological assay and various values of the number of control and case sera in the validation data. (A) & (C) The true distribution of the control and case sera is gamma. (B) & (D) the true distribution of the control and case sera is a truncated normal, we have used a mean and variance that is equal to the mean and various of the gamma distribution for the different AUC-ROC values. For panel (A) and (B) we have fixed the number of control and case sera in the validation set to 5000 each. For panel (C) and (D) we have fixed the AUC-ROC value to be 0.9.

At present the manuscript notes that one can test to see whether K=2 or more distributions are present in the data, which the authors note is a form of misspecification testing. However, this misspecification argument applies only to the mixture cardinality. A key question is whether the distributions themselves can be mis specified without a substantial loss of performance. As an example, the distributions of FACS-derived responses from a luminex/multiplex platform are not gamma distributed. Another example comes from the Santa Clara / Stanford SARS-CoV-2 saga (Bendavid et al), which showed (without intending to) that serological responses for case control samples seem to vary by laboratory. In bother of these cases, modeling with a single gamma distribution would be a misspecification.

Does the theory of mixture models rescue this point? If not, the authors need to make the case that their work will still be valuable. If so, these kinds of broader results from mixture model theory should be introduced and discussed.

Author response: We agree that immuno-assay outcomes can have various distributions. We show in Figure 1 that our improved method can deal with other distributions than the gamma. We have adapted our manuscript to show these broader results and compare them to the cutoff-based methods.

With respect to the lab-to-lab variations, we now propose to either validate the tests in the same lab, or standardize the tests to minimize this source of variation. This is described in the last sentence of the fourth paragraph of the introduction:

If there is significant variation in measures between laboratories, as suggested by Bendavid et al., measurements should be standardized or performed in the same laboratory.

Put more succinctly, the likelihood method does not assume a distribution for the quantitative test measure given infection status ($U|\sigma=0$), yet it seems that a gamma distribution is assumed for all simulations. If the data are simulated from a gamma distribution and then a gamma distribution is used in the model fitting, then the current set of results demonstrate what happens when the model matches the data generating process.

Author response: The adaption of our method described above shows that our method is much broader applicable than only for gamma distributed case and control data. We are grateful to the reviewer for pointing out this issue in our original manuscript, and for inspiring us to provide an improved method.

Finally, if one were to find a mixture of 3 classes, how would one know what the different classes correspond to, if there are only 2 classes in the training data? For the purposes of calculating a seroprevalence estimate, for instance, it would be important to know, a priori, whether some of the middling results were due to cross-reactivity with other (e.g. seasonal) coronaviruses, due to loss of IgG reactivity, or due to mild infection. In other words, the class labels are important. Perhaps the authors meant that the presence of a third group, not represented in the controls, could be tested for, but that interpretation would be difficult to establish?

Author response: We indeed meant to say that we could test for discrepancies between validation and serosurvey data, but the interpretation would be challenging. We have added a sentence about this to the end of paragraph 4 of the discussion in the revised manuscript to clarify this:

“In addition to the severity of infection, many alternative factors could influence the antibody response levels. Most notably, antibody levels have been found to drop over time which can introduce heterogeneities in test measure distributions over time.“

2. That the likelihood-based approach provides superior estimates is not quite established either. The paper focuses on showing that the mixture model provides typically more reliable inferences, i.e. less variable estimates of seroprevalence, and trends. How do the Bayesian estimation procedures compare? Bayesian methods are referred to in the final supplemental figure, but comparisons are not found in the main text.

Author response: This is a valid point. Indeed, we were also interested in comparing the mixture-model estimators more directly to the Bayesian estimation. In the revised manuscript, we have adjusted the figures and main text to add a Bayesian estimation procedure. We compare the mixture-model estimator with the Bayesian estimator proposed by Larremore and others (Larremore, 2020). Unlike the Rogan-Gladen estimator, the Bayesian estimator also takes into account the uncertainty in the sensitivity and specificity of the serological test. For a more detailed description of the method see paragraph 4 of subsection ‘Cutoff-based methods’ of the methods section. Julien Riou, who has advised us on using Bayesian methods in the original manuscript, has performed the additional Bayesian methods in the revised paper, and is therefore a co-author on our revised paper.

The main argument for using the likelihood-based approach (graphically) appears to be found in Figures 1 and 2, based on the distributions of estimates and their biases and variances. The Bayesian methods (which incorporate a likelihood function that includes a cutoff, but does not assume a generating distribution for the data) should be included for comparison.

More broadly, on the point of comparisons, it is widely understood that using "raw" prevalence estimates is a bad (and biased) idea. We suggest removing all results for the uncorrected methods. This will simplify the message and avoid the inclusion of a "straw man" estimates. Fig 1, for instance, would be much more readable without the Max-Youden estimates.

Author response: We have moved the uncorrected cutoff-based methods from Figure 1 to supplementary figure 2 and included Bayesian estimates within Figure 1. To improve the readability of Figure one even further, we have adjusted the range of AUC-ROC values to one that is more realistic for SARS-CoV-2 serological tests (0.8-1).

As an aside, the Bayesian methods that the authors use appear to be estimation procedures, rather than corrections.

Author response: We thank the reviewer for pointing this out and have rephrased the way in which we refer to the Bayesian framework, estimation method, or estimator throughout the revised paper.

Reproducibility:
- The R code cannot be found online or in the manuscript's materials.
Author response: We apologize for not providing this before, all code is available here: <https://gitlab.ethz.ch/jbouman/pist>.
Discussion or broader questions:
- Recent analysis of SARS-CoV-2 serological data suggest that IgG responses wane over time. No changes are needed to the manuscript, per se, but this point may be valuable to discuss in future submissions, particularly given the K=3 mixtures.
Author response: We have added a sentence about this to the end of paragraph 4 of the discussion in the revised manuscript to include a broader discussion on this.
- Issues with learning distributions for positives from only the symptomatic/confirmed cases sounds like an overfitting problem--the lessons from the case control training data do not generalize to the population, in other words. This language may or may not be valuable for bringing in intuition for the ML types out there.
Author response: We adjusted our language to improve intuition in our writing.
- Paragraph 3 of introduction could be updated to reflect some of the numerous recent developments.
Author response: We have updated the introduction to include recent references. The recent references did not change the main messages of the introduction.

Other:
- Page 8 mentions one-step Bayesian model incorporating uncertainty from test validation into prevalence estimates, but no citations are provided. These references appear to do exactly that.
- Gelman, Carpenter. Bayesian analysis of tests with unknown specificity and sensitivity. medRxiv, 2020.­­­­
- Larremore, Fosdick, Zhang, Grad. Jointly modeling prevalence, sensitivity and specificity for optimal sample allocation. bioRxiv, 2020.

Author response: Thank you for drawing our attention to these references, we have read and included them.
- Any analysis of real data would do a lot to strengthen the claims of the authors here.

Author response: We agree that a validation of our method with real data would strengthen our study. We have extensively searched for publicly available data that would allow us to apply the methods we discuss, but did not find any. We are currently setting up collaborations to get access to such data. These collaborations, however, are well-beyond the scope of our present paper.

- Figures could use clarification. Font sizes were small (Fig 2), and line widths or dot sizes made some plots very hard to read (Fig 2), relative to grid lines, for instance.
- e.g. weird fonts Fig 6A y axis?
- Fig 4B... are the x-axis values 75, 100, 150, but presented for each color? Or do the values interpolate between 75, 100, 150, and so on? My guess is the former, but this is a little unclear.
- What are the subplots of Fig1? No notes in caption.
- Various formatting issues in the references, FYI. For instance {SARS-CoV-2} is often uncapitalized.

Author response: Thank you for pointing these issues with the figures, we have incorporated all of them.

**Additional Revisions – not based on reviewer comments**

Besides the adjustments described above, we have made some small adaptations based on our own assessment.

First, we realized that it is difficult to define whether an infection is mild or severe. Moreover, the definition is likely to differ between studies. Therefore, we have decided to use asymptomatic cases in our example of a third distribution of case sera.

We included Figure 4C and D to show the correlation between the number of individuals in the serosurvey and in the validation data. We find it interesting to see how the number of individuals in the validation data can compensate a relatively small sample in the serosurvey and the other way around.

Figure 5B now also shows the total cumulative incidence estimate in addition to the estimate of the cumulative incidence of the asymptomatic and severe cases.

# References

Larremore, D. B. (2020). Jointly modeling prevalence, sensitivity and specificity for optimal sample allocation. *bioRxiv*.
