# Supplementary material for: Estimating the cumulative incidence of SARS-CoV-2 with imperfect serological tests: Exploiting cutoff-free approaches
Source: PLoS Comput Biol. 2021 Feb 26;17(2):e1008728. doi: 10.1371/journal.pcbi.1008728 (PMC7946301; doi:10.1371/journal.pcbi.1008728)
Supplement: Attachment — Submitted filename: ReplyToReviewers_jan_2021-rrr.docx [file pcbi.1008728.s004.docx]

# **Point-by-point replies to the Editor and the Reviewers**

We would like to thank the reviewers for the time and effort they have invested in reviewing our paper for the second time. We have included their comments into the manuscript. Below, we provide point-by-point responses to the reviewers’ comments in blue. In red, we present relevant parts of the revised manuscript.

## Editors’ Comments to the Authors:

Based on the reviews, we plan to accept this manuscript for publication; however, we first ask that you attend to the final comments generously provided by the reviewers. Most of them seem very straight-forward to address, and will enhance the clarity of the published paper.  You will note that one reviewer is skeptical about the new title and framing in terms of cumulative incidence; I understand and support the argument that you put forward in the cover letter for your resubmission, but it seems they did not get this perspective from the manuscript itself.  You might consider adding a sentence or two of discussion about the distinction between cumulative incidence and seroprevalence, and how your model relates to each of them.

Thank you very much for this suggestion. We have added the following section to the beginning of the Discussion to clarify this issue:

‘Throughout this paper, we refrained from referring to sero-prevalence for two reasons. First, sero-prevalence is the frequency of sero-positive individuals in a population and hence inherently coupled to cutoffs. Focusing on the distribution of antibody levels without dichotomizing into sero-positives and -negatives makes it more challenging to determine sero-prevalence. Second, serological tests are usually validated against pre-pandemic sera and sera from individuals with confirmed infection, and not against sera that do or do not contain pathogen-specific antibodies. As a consequence of this choice of positive and negative controls, false positives are individuals that have specific antibodies despite not having been infected, and not individuals for which the test wrongly shows they habor specific antibodies while they actually do not. Correcting for false positives and negatives by both cutoff-based and mixture model methods therefore results in an estimate of cumulative incidence rather than of sero-prevalence. This distinction between sero-prevalence and cumulative incidence is common in epidemiological studies (see for example a metastudy on the cumulative incidence of the 2009 influenza pandemic by Van Kerkhove et al 2013).’

## **Comments from the reviewers**

Reviewer #1: The revised version of the manuscript includes some great improvements, and I only have two minor suggestions.

Page 2 paragraph 3: It might be confusing to refer to a single specific Bayesian application as "the Bayesian framework", given that Bayesian frameworks can be applied to any system or situation. I understand that this is how the authors choose to refer to it within the context of the study, but this is not obvious from the text. A simple change to "we here refer to as .." would probably help to make this clear. Same thing for mixture model in the following paragraph.

We would like to thank reviewer one for pointing this out and have included this suggestion into the introduction.

Table S1 is a great addition, I suggest moving it to the main text if there is space.

Thank you very much for this suggestion. We moved the table into the main manuscript.

**Reviewer #2**: Bouman et al have submitted a revision to their manuscript called "Estimating seroprevalence with imperfect serological tests: exploiting cutoff-free approaches." The manuscript is interesting, and their revision makes improvements upon the first submission. Actually, to call this just a "revision" would be to undersell the big changes that the authors have made across the paper and code. In short, I think the manuscript is a lot stronger, ties in nicely with existing literature, but makes its own contribution clear. This work is likely to be of interest to those who are working with SARS2 ELISA (and other) serological data, and I therefore recommend publication.

In particular, I want to thank the authors for moving beyond the gamma distributions that were used in the first manuscript to try to be more general. I can see that this was a lot of work, and appreciate the effort.

We would like to thank reviewer 2 for these encouraging comments.

The manuscript could be accepted as is, but I also have a few suggestions, which are minor:

1. In the introduction, "the post-hoc Rogan-Gladen correction" is referred to as if readers will know it by name. A slightly more gentle introduction to what the correction is, or is meant to do, might be nice, as context.

We have introduced a more intuitive introduction of the Rogan-Gladen correction in the introduction.

‘More specifically, Rogan and Gladen describe a post-hoc correction that essentially subtracts the expected number of false positives and adds the expected number of false negatives to the binomial estimator.’

2. Page 3 "specificty" typo.

This has been fixed. Thank you for noticing.

3. Page 5 "a certain statistical power of (...) decreases" This sentence doesn't read well without the (parenthetical).

We have replaced this sentence with:

‘Additionally, we find that, with increasing test accuracy, the number of control and case sera required to obtain a statistical power of 0.9 decreases.’

4. Given the rapid acceleration of cases across much of the northern hemisphere, the end of paragraph 1 in discussion should suggest that this will be of value specifically for *early pandemic* SARS-CoV-2 serosurveys, since seroprevalence is likely to be higher.

We have added *early pandemic* to this sentence.

5. Finally, I wasn't quite sure that I understood the discussion about the inference of cases that were not included in the validation of the test. Methodologically, how should one do this? (e.g. AIC or BIC or some other model selection / complexity control?) I think the point is made in a nice visual way in Fig 5, but it might be worth at least discussing ways that people could look to see whether their dataset supports a third distribution. By the way, should the title on Fig 5B be slightly different to note that there are asymptomatic controls too?

We describe this in the third paragraph of page 7:

‘We recommend to always use a mixture model with an additional unknown distribution and to test its fit with a likelihood-ratio test against a null model that includes all known case distributions.’

And have also added a sentence about this to the results:

‘In practice, one can test for the existence of a missing case distribution by applying a likelihood ratio test on the extended model and the standard mixture model.’

Text of Fig 5B: Indeed, the title of figure 5B should be different. As the space is limited we have removed the title from all subfigures and believe that the figure legend should be sufficient to understand this figure.

As a final note, I noticed that the title is now about cumulative incidence instead of seroprevalence, but given that there is evidence of (typical) waning antibody titers over time, from some studies, I wonder if perhaps seroprevalence is a more appropriate word than cumulative incidence. I fear that cumulative incidence may not be quite as accessible via serology after nearly a year, as we may have initially hoped. I leave it to the authors and editor to decide, of course.

We apologize for not providing a better explanation for why we decided to change to using ‘cumulative incidence’. We have added an explanation to the beginning of the discussion (see also our reply to the comments of the editor):

‘Throughout this paper, we refrained from referring to sero-prevalence for two reasons. First, sero-prevalence is the frequency of sero-positive individuals in a population and hence inherently coupled to cutoffs. Focusing on the distribution of antibody levels without dichotomizing into sero-positives and -negatives makes it more challenging to determine sero-prevalence. Second, serological tests are usually validated against pre-pandemic sera and sera from individuals with confirmed infection, and not against sera that do or do not contain pathogen-specific antibodies. As a consequence of this choice of positive and negative controls, false positives are individuals that have specific antibodies despite not having been infected, and not individuals for which the test wrongly shows they habor specific antibodies while they actually do not. Correcting for false positives and negatives by both cutoff-based and mixture model methods therefore results in an estimate of cumulative incidence rather than of sero-prevalence. This distinction between sero-prevalence and cumulative incidence is common in epidemiological studies (see for example a metastudy on the cumulative incidence of the 2009 influenza pandemic by Van Kerkhove et al 2013).’

In sum, despite a few minor suggestions, the work by Bouman et al is a valuable contribution to the serosurvey literature. Its blend of practical application, theoretical development, and a commitment to open-source tools make it a great fit for PLOS Computational Biology. I recommend publication.
